# The Importance of Reporting Energy Values of Human Milk as Metabolizable Energy

**DOI:** 10.3389/fnut.2021.655026

**Published:** 2021-07-07

**Authors:** Tanis R. Fenton, Seham Elmrayed

**Affiliations:** ^1^Community Health Sciences, Cumming School of Medicine, O'Brien Institute of Public Health, Alberta Children's Hospital Research Institute, University of Calgary, Calgary, AB, Canada; ^2^Nutrition Services, Alberta Health Services, Calgary, AB, Canada

**Keywords:** human milk, breast milk, calories, energy, energy metabolism, calorimetry, breastfeeding, infant

## Abstract

Nutrition science has a convention to report metabolizable energy instead of gross energy. Metabolizable energy at 4 kilocalories per gram for protein and carbohydrate, 9 kcal per gram for fat (kilojoules: 17 and 37, respectively) represents the food energy available for metabolism. However, this convention to use metabolizable energy has not been uniformly applied to human milk. Human milk is often reported as gross energy, which is about 5–10% higher than metabolizable energy. To obtain accurate human milk energy estimates, milk samples need to contain the same proportion of high fat hind milk that an infant obtains.

## Introduction

There are discrepancies in the reports of human milk's energy content. While many textbooks and commercial handouts report specific numerical energy (calorie or joule) values for human milk, there are two main reasons to have low confidence in these numbers. These reasons include the lack of consensus regarding how milk energy should be quantified and reported as well as the difficulty to obtain representative samples of human milk as it is consumed by infants.

## Metabolizable Energy

An important source of error in the estimation of human milk energy is the difference between gross and metabolizable energy. Gross energy is the quantity of combustible energy contained in a food, as measured using a bomb calorimeter. Gross energy values over-estimate the energy available to the infant for metabolism. A common assumption for human milk has been that gross energy values are the correct values (1). However, the convention in nutrition science is to report all foods' energy content as metabolizable energy (2) instead of gross energy.

## Human Milk is Difficult to Sample

Human milk is likely the most difficult food to measure and quantify. It is almost impossible to replicate breastmilk samples as it continually changes in fat and energy content, over a feeding, over each day over time beginning after birth. It is important to sample milk to replicate the changing composition as an infant feeds numerous times a day (3, 4). Those trying to obtain representative samples of human milk require not only 24-h sampling but also the same degree of breast emptying that an infant achieves and samples throughout each feed. To accurately estimate the energy content, it is important to obtain the same proportions of lower fat fore milk and high fat hind milk as an infant would extract.

## Reasons to Use Metabolizable Energy of Human Milk

There are four reasons to report the metabolizable energy content of human milk instead of the gross energy content (Table 1). First, the convention in nutrition science is to report metabolizable energy because it represents the food energy available for metabolism (2), including for infants. Second, the use of metabolizable energy will align infant metabolism and energy requirements with their diet, providing superior agreement in human milk studies that examine infant metabolism and infant energy requirements (5).

**Table 1:** Reasons to report the metabolizable energy content of human milk.

The convention in nutrition science is to report metabolizable energy of foods
The use of metabolizable energy will align infant metabolism and energy requirements with their diet
It is clinically important to accurately quantify infant energy intake to ensure that infant energy needs are met
The use of metabolizable energy for human milk would avoid a systematic overestimation over the energy content of human milk

Third, it is clinically important to accurately quantify infant energy intake. Metabolizable energy is used to report the energy content of other foods routinely consumed by infants including complementary foods and infant formula, as well as human milk fortifiers and modulars that are used clinically. Given that human milk is considered a nutrition gold standard for infants in their first months of life (making it a widely recommended and frequently used food) it is important to accurately understand the metabolizable energy content of human milk. Fourth, in clinical settings when gross energy values are used with over-estimated human milk energy intakes, clinicians may not optimize energy intakes if they believe energy needs are met. The use of metabolizable energy for human milk would avoid a systematic overestimation error between the energy content of human milk. A systematic error is one that affects results consistently in the same direction.

## How are Metabolizable Energy Values for Human Milk Obtained?

There are two methods to obtain metabolizable energy values for human milk. Metabolizable energy can be estimated by multiplying each energy containing macronutrient by their Atwater factor or by lowering gross energy values by agreed upon values. Gross energy estimates could be reduced to estimate metabolizable energy, based on the milk composition.

## Atwater Factors

Atwater and colleagues over 100 years ago conducted detailed experiments of numerous foods to quantify the metabolizable energy content of foods. Their work was endorsed and reinforced in 1973 by the American Department of Agriculture (6). The Atwater factors have since been endorsed by the Food and Agriculture Organization (2) and numerous countries (7–9). The Food and Agriculture Organization recommends using metabolizable energy to report the energy content of foods since nutrient requirement estimates are based on actual energy expenditure estimates (2). The Atwater figures representing metabolizable energy have become the convention for reporting food energy for foods in food composition tables, with one exception, and that is human milk. The primary reason for a lack of convention for human milk energy quantification is likely primarily because this issue is not well-known and to date no decision has been made by milk researchers.

Scientists and dietitians are familiar with the Atwater factors that represent the energy content of foods, that is 4 kilocalories (kcal) per gram for protein and carbohydrate and 9 kcal per gram for fat (kilojoules: 17 and 37, respectively). Perhaps not widely known is that these Atwater factors represent metabolizable energy values. In contrast, the gross energy factor of 5.65 for protein may not be as well-known. The difference between gross and metabolizable energy is the energy that is not bioavailable for metabolism. There are three reasons some food energy is not bioavailable; since not all nutrients are fully digestible (e.g., dietary fiber), absorption of the energy nutrients is not 100% and some urinary energy excretion is obligatory. Of the macronutrients, protein has the greatest difference between gross and metabolizable energy, equal to a 30% difference between 5.65 and 4.0 kcal/gram (10). The reason that protein has the greatest difference between gross energy and metabolizable energy is that there is an energetic cost to excrete nitrogen released during protein turnover and catabolism, which is excreted after conversion to urea. The differences between gross and metabolizable energy for fat and carbohydrate are mainly related to imperfect absorption, so are ~2%, which is considerably smaller than they are for protein (10).

The Food and Agriculture Organization recommends using 2 kcal/gram for the energy content of fermentable fiber (2), which is likely applicable to human milk oligosaccharides as they are not readily digestible by infants and some of them are fermented in an infant's intestine. At an average of 1.8 g/100 mL, oligosaccharides make up about 22% of the carbohydrates in human milk (1). Using the Food and Agriculture Organization recommended 2 kcal/gram for the energy content of fermentable fiber human milk oligosaccharides likely contribute about 7 kcal/100 mL. When lactose is measured and oligosaccharides are ignored, human milk energy will be underestimated by the energy of the oligosaccharides.

Human milk protein and energy would be over-estimated if nitrogen is measured instead of actual protein and then assumed to represent protein (10).

While the differences between gross and metabolizable energy likely create important differences in human milk estimates, the values for both quantities of energy have not been thoroughly studied. One example of a study that quantified both gross and metabolizable energy was the work by Thomas et al. (11). They quantified energy of human milk of 20 mothers of preterm infants between 14 and 18 days postpartum at 71 kcal/100 mL gross energy and at 6–8% less (66 kcal/100 mL) metabolizable energy (11).

Reilly et al. proposed reducing gross energy estimates to 93% for estimates of metabolizable energy on the basis of Southgate and Barrett's 1960's work on human milk metabolism (12) and metabolism of adult mixed diets (5). This value may be the conversion value that is needed, or the conversion value might need to be variable as the composition of human milk changes in the first lactation weeks (1). More work is needed, including a systematic review of existing data to establish conversion factors between gross energy and metabolizable energy. Considering Reilly's et al. proposal of 93% (5) and the study that reported both (11), gross energy estimates likely overestimate human milk's energy content by 5–10%.

While it might be tempting to think that metabolizable energy values of human milk should be adjusted for postnatal age and infant ability to absorb nutrients, there is no convention in nutrition science to alter food energy estimates based on variable absorption by clinical condition or dependent on some other variable such as age (10).

## Conclusions

To improve understanding of human milk energy composition there is a need for systematic reviews to summarize the reported data, that is establish the differences between the metabolizable vs. gross energy values. There is also a need for the establishment of conventions for the use of metabolizable energy and for the use of superior milk sampling methods.

## Author Contributions

TF wrote the first manuscript draft. SE and TF edited the manuscript and approved the final submitted version.

## Conflict of Interest

The authors declare that the research was conducted in the absence of any commercial or financial relationships that could be construed as a potential conflict of interest. The handling editor declared a past co-authorship with the author TF.

## References

[1] Gidrewicz DA Fenton TR. A systematic review and meta-analysis of the nutrient content of preterm and term milk. BMC Pediatr. (2014) 14:216. 10.1186/1471-2431-14-216 25174435 PMC4236651

[2] Chen J Hevassus-Agnes C Gilani GS. Food and Agriculture Organization of the United Nations. Food Nutrition Paper. 77: Food energy Annex I: participants—technical workshop on food energy: methods of analysis and conversion factors (2003). Available online at: https://www.sennutricion.org/media/Docs_Consenso/Food_energy_methods_of_analysis_and_conversion_factors-FAO_2002.pdf (accessed January 8, 2021).

[3] Saarela T Kokkonen J Koivisto M. Macronutrient and energy contents of human milk fractions during the first six months of lactation. Acta Paediatr. (2005) 94:117681. 10.1111/j.1651-2227.2005.tb02070.x 16203669

[4] Kent JC Mitoulas LR Cregan MD Ramsay DT Doherty DA Hartmann PE. Volume and frequency of breastfeedings and fat content of breast milk throughout the day. Pediatrics. (2006) 117:e38795. 10.1542/peds.2005-1417 16510619

[5] Reilly JJ Wells JCK. Duration of exclusive breast-feeding: introduction of complementary feeding may be necessary before 6 months of age. Br J Nutr. (2005) 94:869–72. 10.1079/BJN20051601 16351760

[6] Merrill A Watt B. Energy Value of Foods, Basis and Derivation. USDA Agricultural Handbook No. 74. Washington, DC: Human Nutrition Research Branch (1973).

[7] Canadian Food Inspection Agency. Food Labelling for Industry. Available online at: https://www.inspection.gc.ca/food-label-requirements/labelling/industry/nutrition-labelling/elements-within-the-nutrition-facts-table/eng/1389206763218/1389206811747?chap=1. 2018 (accessed January 8, 2021).

[8] Australia New Zealand Food Standards Code. Schedule 11: Calculation of Values for Nutrition. (2015). Available online at: https://www.legislation.gov.au/Details/F2018C00367 (accessed January 8, 2021).

[9] Food Drug Administration, USA. Nutrition Labeling of Food. Part 101.9. (2020). Available online at: https://www.accessdata.fda.gov/scripts/cdrh/cfdocs/cfcfr/CFRSearch.cfm?fr=101.9 (accessed January 8, 2021).

[10] Fenton TR McLeod G. Direct measurement and estimation of the energy content of human milk. In: O'Connor D McGuire M, editors. Human Milk: Sampling, Measurement, and Content of Energy-Yielding Nutrients and Other Macromolecules. London: Elsevier (2021). p. 175–90. 10.1016/B978-0-12-815350-5.00007-3

[11] Thomas MR Chan GM Book LS. Comparison of macronutrient concentration of preterm human milk between two milk expression techniques and two techniques for quantitation of energy. J Pediatr Gastroenterol Nutr. (1986) 5:597–601. 10.1097/00005176-198607000-00016 3735009

[12] Southgate DA Barrett IM. The intake and excretion of calorific constituents of milk by babies. Br J Nutr. (1966) 20:36372. 10.1079/BJN19660036 5952575

